# Healthcare professionals’ perceptions of community-based rehabilitation in KwaZulu-Natal, South Africa

**DOI:** 10.4102/phcfm.v13i1.2461

**Published:** 2021-01-27

**Authors:** Sithembiso B. Blose, Sudipa Doeraj, Sabiha Padia, Kaveshan Pillay, Kinita Reddy, Verusia Chetty

**Affiliations:** 1 Department of Physiotherapy, Faculty of Health Sciences, University of KwaZulu-Natal, Durban, South Africa

**Keywords:** Community-based rehabilitation, healthcare professionals, people with disabilities, KwaZulu-Natal, CBR Worker

## Abstract

**Background:**

People with disabilities (PWDs) continue to experience challenges with access to healthcare. Community-based rehabilitation (CBR) is an approach that advocates for equal opportunities and social inclusion of PWDs to enhance their quality of daily life. Healthcare professionals are crucial in the implementation of CBR. However, little is known about the perception of healthcare professionals on this approach to rehabilitation in South Africa.

**Aim:**

This study sought to explore perceptions of healthcare professionals on CBR in the province of KwaZulu-Natal, South Africa.

**Setting:**

This study was located across four public healthcare facilities spanning districts to tertiary levels care in KwaZulu-Natal, situated in rural and peri-urban areas.

**Methods:**

An explorative qualitative approach using focus group discussions was used to collect data from healthcare professionals employed at these public hospitals in the province. Twenty-five healthcare workers participated in four focus group discussions, with four to eight participants per group. Data were transcribed and analysed using thematic analysis.

**Results:**

The findings revealed four dominant themes, namely, the CBR conundrum, CBR enablers, perceived impediments to CBR implementation and a proposal for the implementation of CBR.

**Conclusion:**

Continual promotion of, as well as education and training on, CBR for healthcare professionals, was understood as an imperative for the development and roll-out of CBR programmes in South African communities. Excellent communication about CBR programmes was described as key to ensuring social inclusion, quality of life and access to services for PWDs.

## Introduction

Community-based rehabilitation (CBR) is a strategy to enhance the quality of life for people with disabilities (PWDs) ‘through equalisation of opportunities, poverty reduction, and social inclusion by improving access to essential human rights services’. It is implemented through a combined effort of stakeholders, which include PWDs and their families, communities and governmental and non-governmental organisations.^1^ Community-based rehabilitation was established in 1981, following the Alma Ata declaration, as an approach for improving access to health and rehabilitation services, especially in rural and underserved communities.^2^ A CBR matrix was developed to guide the implementation of CBR, following the United Nations Convention on the Rights of Persons with Disabilities (CRPD). The matrix consists of five domains, namely health, education, livelihood, social and empowerment, aimed at enforcing the rights of PWDs.^3^ Each domain has five themes that guide the implementation of CBR. Community-based rehabilitation had been reported to be implemented in over 90 countries, but most of the programmes focus on one or two domains, mostly health and education.^4^ The health domain includes promotion, prevention, medical care, rehabilitation and assistive devices as underlying themes, whilst the education domain includes early childhood; primary, secondary and higher; non-formal and lifelong learning. Despite much focus being placed on the health and education domains, PWDs continue to experience challenges in accessing healthcare, rehabilitation and education, contributing to significantly low levels of employment, thereby rendering them trapped in a poverty cycle.^5^

It is estimated that 1 billion (15%) of the world’s population have disabilities, and of these, 80% are from low- and middle-income countries (LMICs).^6^ Fifty to fifty-eight million (5% − 17%) PWDs live in sub-Saharan Africa.^7^ It is estimated that the prevalence of disability is 7.5% in South Africa; however, this figure is treated as an underestimate, because PWDs living in institutions, children under the age of 5 years and persons with psychosocial and certain neurological impairments were not included because of data limitations.^8^ A 2016 community survey, using three severity cut-off/threshold broad measurements, found that the disability prevalence is 16%, when compared with 17% in the 2011 census.^9^ People with disabilities are also amongst the poorest and least empowered community members, especially in LMICs.^2^ Understanding of the needs and challenges faced by PWDs remains a mammoth task for the majority of healthcare professionals.^10^ Therefore, PWDs should be engaged in the planning and implementation of CBR services, as they are vital stakeholders who offer great insight into feasible approaches to improve ‘their’ quality of life and livelihoods.

The accomplishment of CBR programmes should be actualised through a multisectoral and multidisciplinary approach advocating for combined efforts of PWDs, their families, communities, governmental organisations and non-governmental organisations.^11^ Poor coordination, collaboration and teamwork will create barriers to the successful implementation of CBR, influencing the effectiveness and sustainability of CBR projects and programmes.^11,12^ Amongst the role players are healthcare professionals who play a fundamental role in the implementation of CBR.^3^ These healthcare professionals include medical officers, therapists, social workers, dieticians, nurses and pharmacists. There is a strong argument that CBR implementation in some regions is still based on the medical model of disability.^13^ The medical model focuses on diagnosis and treatment. This model may diminish the quality of life of an individual as it also aims at ‘correcting’ the disability. It has less consideration of the social issues or aspect of a person.^14^ Therefore, healthcare professionals are seen as experts, which often renders the opinions and concerns of PWDs less important in some settings.^15^ The role of a healthcare professional is critical in the implementation of CBR but not the dominant role.

The healthcare professional needs to be skilled as an advocate, communicator, professional, collaborator, leader and scholar – as these are all necessary as core competencies. As a health advocate for PWDs and CBR, healthcare professionals can begin to play their role as facilitators, rather than experts in the field of disability.^16^ Therefore, healthcare professionals as health advocates are expected to combine their professional knowledge with advocacy to improve the implementation of CBR.^17^ Other core competencies of healthcare professionals, such as those of a collaborator and a leader, are required to strengthen collaboration with stakeholders in the implementation of CBR. This collaboration will facilitate necessary changes for disability-inclusive programmes that will influence implementation, training and policy, which demand that stakeholders keep abreast of the latest knowledge.^18,19^

Therefore, the need to retrain healthcare professionals on CBR, as identified by the National Rehabilitation Policy (NRP), becomes a critical strategy for CBR implementation (NRP).^20^ The retraining will enhance CBR implementation by improving awareness and understanding of CBR amongst healthcare professionals. However, the NRP was replaced by the Framework and Strategy for Disability and Rehabilitation in South Africa (FSDR) in 2015. Still, there is no indication of any training being conducted, although the FSDR also mentions CBR as a platform for service delivery.^21^ An awareness and understanding of CBR enable healthcare professionals to take into account the voices of PWDs and allow for better engagement with the community, thereby improving the participation and social inclusion of PWDs.^15^

Awareness and understanding of CBR will most likely lead to attitude change, strengthening of family relationship with PWDs and better stakeholder engagement. Stakeholders play a pivotal role in collaboration for the implementation of CBR programmes that are based on rights of PWDS.^11^ Community-based rehabilitation stakeholders are expected to have up-to-date knowledge of CBR through continuous training. Training institutions and academics have been found to be a critical source of knowledge. Still, there is a challenge of transferring this knowledge and skills to all stakeholders, including healthcare professionals.^19^ The lack of awareness and understanding of CBR by healthcare professionals and the evidence that training for healthcare professionals is deficient contributes to the misunderstanding of the purpose of CBR.^11,19,21,22^ The lack of understanding and awareness of CBR amongst healthcare professionals can indirectly lead to negative attitudes and the disempowerment of PWDs by healthcare professionals.^21^ Therefore, the purpose of this article was to explore the perception of healthcare professionals in KwaZulu-Natal on CBR to understand their experience and address the gaps in the implementation of successful programmes in our communities.

## Research method and design

### Study design

This study used a qualitative design to explore perceptions of healthcare professionals on CBR in KwaZulu-Natal.^23^ This approach allowed for an in-depth understanding of experiences and knowledge of healthcare professionals regarding the planning, implementation and sustainability of the CBR.

### Study location

This study was located across four public healthcare facilities spanning district to tertiary levels of care. Healthcare professionals, registered by the Health Professions Council of South Africa and permanently employed, were purposively sampled from each hospital. Variation of the sample population was achieved through the inclusion of male and female participants with diverse experience in their respective professions and different ethnicity.

Recruitment of healthcare professionals was conducted at hospitals in KwaZulu-Natal. The hospitals identified were those linked to the University of KwaZulu-Natal community-based primary healthcare training platform.^24^ This study involved two district hospitals, two hospitals with district regional and tertiary services. The study sites are situated in rural and peri-urban areas of the province, within a radius of 300–400 km.

### Study population and sampling strategy

Twenty-five healthcare professionals were recruited from the selected hospitals by convenience sampling. Permission was obtained from the hospital authorities as part of the recruitment strategy. The participants were then recruited verbally followed by written information about the study. The healthcare professionals included physiotherapists, speech and language therapists, occupational therapists, audiologists, dieticians, pharmacists, dental therapists, psychologists, social workers, nurses and medical doctors who were qualified healthcare professionals registered with a recognised health board or council in South Africa and who were employed by the KZN Department of Health. A multidisciplinary team was necessary; as such team dynamics are required for effective implementation of CBR.

### Data collection and data analysis

Four focus group interviews were conducted at the respective hospitals in English. Focus groups had between four and eight members participating in the discussion. Open-ended questions were used to create an opportunity for an in-depth conversation on knowledge of CBR, training on CBR and role in CBR implementation. Probing and clarification were performed to gain a full understanding of comments and responses during the interview. Interviews lasted approximately 30–60 min and a voice recorder and notebook were used to record verbal and non-verbal responses during the interviews. The recordings were transcribed verbatim immediately after interviews with the researchers and immediately analysed thematically to identify emerging themes and sub-themes.^25^

### Trustworthiness and rigour

The transcribed audio recordings were checked for any missing data against the audio recordings by the researchers and a moderator to improve trustworthiness. Data were read and re-read for familiarisation by two independent researchers to obtain an in-depth understanding of its content. Researchers discussed the themes at length until a consensus was reached. Significant quotes were highlighted and patterns were coded. An expert in qualitative research approaches, employed at the tertiary institution, was used to moderate codes as well as processes independently.

### Ethical consideration

Ethical approval was obtained from the Humanities and Social Sciences Research Ethics committee of the University of KwaZulu-Natal (Ethical clearance number: HSS/0198/019U). Gatekeeper, including KwaZulu-Natal’s Department of Health (DOH) Research and Knowledge Management, approval for this study was obtained before commencing with the data collection (KZN DOH ref. number: KZ 201909 021). Healthcare professionals signed informed consent to participate voluntarily, with no incentives being offered during this study. Participants were informed of their right to withdraw from this study at any time, should they wish to do so. Confidentiality and anonymity were maintained throughout the study process.

## Findings

The focus group discussion explored the awareness and knowledge of CBR of healthcare professionals. Participants included six male and 19 female healthcare professionals. Eleven of the healthcare professionals had < 5 years of working experience in the Department of Health, whilst 14 professionals had more than 5 years of working experience. Healthcare professionals who participated in this study included medical officers (three), a pharmacist (one), a physiotherapist (11), occupational therapists (three), speech and language therapists (two), audiologists (two), a social worker (one) and dieticians (two) (refer to Table 1).

**TABLE 1:** Demographics of participants.

Profession	Gender	Work experience (years)	Level of care	Identification number
Medical officer	Female	01	Regional	MO1
	Female	02	Regional	MO2
	Female	04	District	MO3
Pharmacist	Female	02	Tertiary	PH1
Physiotherapists	Female	08	Tertiary	PT1
	Male	20	Tertiary	PT2
	Female	01	Regional	PT3
	Female	07	Regional	PT4
	Female	14	Regional	PT5
	Female	01	Regional	PT6
	Male	03	District	PT7
	Female	01	District	PT8
	Male	01	District	PT9
	Male	04	District	PT10
	Female	01	District	PT11
Occupational therapists	Male	11	Tertiary	OT1
	Female	02	Tertiary	OT2
	Female	01	District	OT3
Speech therapists	Male	07	Regional	SP1
	Female	12	Tertiary	SP2
Audiologists	Female	07	Regional	AU1
	Female	09	Tertiary	AU2
Social worker	Female	13	Tertiary	SW1
Dietician	Female	01	District	DT1
	Female	06	Tertiary	DT2

The discussions with healthcare professionals yielded four themes. These overarching themes included the CBR conundrum, CBR enablers, perceived impediments to CBR implementation and a proposal for the implementation of CBR.

### The community-based rehabilitation conundrum

The theme ‘the community-based rehabilitation conundrum’ refers to the phenomenon around challenges that riddle healthcare professionals’ understanding of the strategy. Community-based rehabilitation is a complex approach and requires extensive understanding in order to effectively implement it in a feasible context-specific strategy. The current study found that the misconception of CBR by healthcare professionals is dominant. These misconceptions and interpretations of CBR will inadvertently lead to ineffective implementation of strategies.

Some healthcare professionals believed that CBR is synonymous with home-based therapy. Their voices are reflected in the below quotes:

‘CBR is basically taking treatment out to areas that cannot access proper medical care. That is my understanding of CBR. Taking out our treatment to rural areas to people who find it hard to access care in the cities.’ (Physiotherapist 5, regional hospital)

‘There are still a lot of people who are disabled or need rehabilitation that are not getting it. Also, even for us to go to their homes for, like an hour, just to give them a bit of therapy. It’s just therapy at your home.’ (Audiology 2, regional hospital)

Other participants believed CBR to be an extension of healthcare services for patients who have been discharged from the hospitals. This is reflected in the illustrative quotes below:

‘We usually discuss it in the MDT when there are clients who require CBR. When you are treating the patient in the hospital, the patient is coming from a family, from a community, so as a pre-discharge plan we have to look into where is the patient going to when they are discharged. What kind of support are they going to get at home?’ (Social Worker 1, tertiary hospital)

‘It is taking the health care professionals out of the hospital setting, and getting them to go into the patients home and real lives and incorporate their treatment and rehabilitation programmes to be in tune with the community.’ (Physiotherapist 9, district hospital)

However, some of the healthcare workers believed that CBR was a novel concept challenging the essential interpretation and possibly the implementation of the strategy:

‘[*N*]o, it’s a new concept, so I don’t really know what it will entail.’ (Dietician 2, tertiary hospital)

‘[*N*]o, I haven’t heard about it (referring to CBR).’ (Medical Officer 3, district hospital)

One of the healthcare professionals believed that she had basic knowledge, but the following quote highlights her explanation and exposes the level of her understanding:

‘[*A*] holistic approach to health. It’s seeing that the determinants of health are environmental, social as well as physical, and it’s trying to target areas where health can be supported, and it’s got a couple of different tiers that you use for that, in order to make it work.’ (Occupational Therapist 3, district hospital)

Another misinterpretation of CBR is reflected in a medical officer’s suggestions:

‘[*T*]here are a couple of challenges that may exist which need to be explored, being the commuting of our rehabilitation team to the community where proper structures and provisions should be made to ensure the rehab(ilitation) team gets to the community safely; as well as once the rehab team is there, they need to ensure safety while they are there. Secondly, making sure that there is equipment on hand and that the rehab(ilitation) team has a full set of equipment, which they need. To ensure there is a set standard in which the services given to the community is adequate to that of the hospital.’ (Medical Officer 2, regional hospital)

This narrative makes it clear that the medical officer understands CBR as a continuity of rehabilitation in the community and refers to providing adequate resources to facilitate such care.

### Community-based rehabilitation enablers

The second theme ‘community-based rehabilitation enablers’ refers to facilitators for effective CBR that healthcare workers identified during the discussions.

A medical officer said ‘looking into the name, you see it’s supposed to be comprehensive healthcare where it will include the MDT and stakeholders involved in health’ (Medical Officer 1, regional hospital), identifying that collaboration amongst key stakeholders and role players within health and the community will contribute positively to the implementation of CBR.

A physiotherapist also believed that:

‘[*S*]ome of the challenges are, firstly, in identifying key role players in the community that are willing people; so it’s like getting people on board in terms of your rehabilitation programme and how you are going to implement it, and there is really key players to every section of that implementation.’ (Physiotherapist 11, district hospital)

This was seen as a critical step in involving essential and willing role players into CBR programmes and development.

As much as healthcare professionals are regarded as specialists in their fields and CBR requires a cadre of such professionals for its implementation, the healthcare professionals in this study also believed that community healthcare workers (CHWs) were key role players for the successful implementation of CBR. This is clearly reflected in the below illustrative quotes:

‘[*T*]hey have the community healthcare workers, of which it’s them who normally go before the community service workers to help them with identifying whatever they need or identify the homes or patients who need to be assisted, especially with rehabilitation. They will be referred to us, or the community service therapist will go to them to intervene and take care of patients’ needs or refer to other disciplines besides the rehabilitation team. They could assist with CBR.’ (Physiotherapist 4, regional hospital)

‘[*I*]t is very beneficial as they do not have to come so far to get these services. The community healthcare workers can help with that. Therefore, CBR is quite a great thing as it serves the need of people with disabilities.’ (Physiotherapist 7, district hospital)

‘[*T*]he patient doesn’t come to the hospital, or they can’t come to the hospital, so it makes it easier for them to access the services here with the help of the community healthcare workers.’ (Speech therapist 2, tertiary hospital)

### Perceived impediments to community-based rehabilitation implementation

Whilst CBR was regarded as a good strategy, perceived impediments were identified, which were believed to pose barriers to the implementation of CBR. These challenges included lack of resources, poor translation of CBR theory into practice, lack of continuous training and poor safety and security.

The lack of resources, which include personnel and physical resources, was regarded as a critical impediment to CBR implementation that was seen to be a frustration to healthcare professionals:

‘[*I*]t does not actually happen at hospitals. There is no staff for that for us to go to do CBR because we are short-staffed and due to lack of transport and money.’ (Audiologist 2, tertiary hospital)

‘[*T*]here are transport issues; not enough staff; lack of man-power; lack of financial support. The challenges have become much worse over the last ten years.’ (Occupational Therapist 1, tertiary hospital)

‘[*W*]e do not implement CBR due to issues like being short-staffed and transport problems.’ (Physiotherapist 1, tertiary hospital)

There was an acknowledgement of the existence of CBR policies and guiding documents. However, the participants felt that the availability of CBR policies does not necessarily translate into practice, and limited or no support is provided by governing structures:

‘If you are going to put up a policy, put up a structure for it. Put up the groundwork, foundation for it. If the foundation is there, it will stand up, and it will work. But most of the policies get implemented at university level first or academic hospitals.’ (Audiologist 1, regional hospital)

‘On paper, it’s a good idea, but implementation-wise it’s a bit difficult to do.’ (Speech therapists 1, regional hospital)

‘It’s not something that the management or the Department of Health is aware of, or it is just one of those policies that are there but is not implemented on the ground level.’ (Audiologist 2, tertiary hospital)

Some healthcare professionals indicated that the only training on CBR received was at the university level and there has been no training where they are based. Healthcare professionals felt that they were inadequately trained on CBR and its roll-out creating barriers to CBR programme.

‘[*A*]ll the training we had was more at the university level rather than at the hospital level. There hasn’t really been any additional training that we’ve received post-varsity.’ (Occupational Therapist 2, tertiary hospital)

‘[*H*]ealthcare professionals aren’t trained enough to be able to implement it (CBR), and even when we are, we’re too constrained by our clinical settings and by the requirements of government, so even though they (government) support CBR, they tell us to work in particular ways.’ (Occupational Therapist 1)

The influence of crime and personal safety with regard to the healthcare professionals going out into the communities to implement CBR was a common impediment voiced by healthcare professionals:

‘[*S*]ome places were not being served due to hijackings.’ (Occupational Therapist 2, tertiary hospital)

‘[*Y*]ou have to think about whether the area you will be going to is safe.’ (Physiotherapist 3, regional hospital)

‘[*I*] want to think that resources are always a problem. First of all, we have to be transported there and want to think it is a safe place to work, considering the social problems we face in our communities.’ (Medical Officer 2, regional hospital)

### A proposal for the implementation of community-based rehabilitation

The healthcare professionals made suggestions regarding the implementation of CBR, including promoting awareness about CBR amongst communities, obtaining greater community involvement and using available resources for CBR roll-out.

An audiologist said:

‘[*T*]he people that we are servicing don’t know what they actually deserve. We know about it, and we are sitting with the knowledge. The people that need this knowledge is the community itself. If they don’t get the awareness of what they are supposed to have, they actually don’t know that they are supposed to access.’ (Audiologist 2, tertiary hospital)

This is important as the community is an essential partner in the development of sustainable CBR programmes. *A physiotherapist also voiced that* ‘more health awareness and promotion should be forced into different communities that we do CBR in’ (PT11).

A dietician suggested that:

‘[*I*] also think making use of the resources that are available in the community. So not coming in from the outside and saying, “this is what we think should be done” and not having a one-size-fits-all for every community. A big focus of ours is identifying who are the community leaders and a big focus on how you try to implement your plan. You cannot come in and act as an expert. You have to come in from the ground level and find influential people within the community who can help you sustain, as well as implement, it.’ (Dietician 2, tertiary hospital)

This belief is two-fold and makes reference to using resources available in the community for CBR and harnessing the voices and contribution of the community members as equal partners.

The benefit of partnering with community members is also reflected in a physiotherapist’s belief that:

‘[*I*]n implementing it (CBR), I think it is trying to get the community together and the people to come together and to actually implement whatever it is you’ve come up with and sustainability because you don’t know whether they are still going to carry on with it, and will it be sustained.’ (Physiotherapist 9, district hospital)

## Discussion

The purpose of this study was to understand healthcare professionals’ perceptions of CBR and its implementation in KwaZulu-Natal, South Africa. This study also explored healthcare professionals’ understanding and knowledge of implementing CBR. These perceptions are important as they influence the approach to the successful implementation of CBR for PWDs by healthcare professionals. However, misconceptions of CBR by healthcare professionals, because of a lack of knowledge and limited awareness, can contribute to poor implementation.^11^ Some healthcare professionals, in this article, displayed a necessary basic insight into the CBR concept, whereas many others lacked understanding of the strategy. Community-based rehabilitation is not merely decentralising care to home settings, which was a common misconception of healthcare professionals, but it involves PWDs, their families and the community at large.^26^

Home-based care is any form of assistance provided to a sick person within their home setting. It is not only merely intended to give care to dependent patients but also for a continuum of care through rehabilitation, psychosocial support, reducing stigma and discrimination, as well as adherence monitoring of people living with HIV (PLHIV).^27^ However, CBR is understood to be an approach and a strategy that is a complex concept because of its nature that seeks to promote social inclusion, improve quality of life and improve access to services.^10^ Although CBR is understood to be a complex concept, healthcare professionals need to have the insight to differentiate between CBR and home-based care.^28^ This approach requires many layers of involvement, from a medical focus to a social paradigm. Healthcare professionals in this study did not make the necessary links with the social aspect of CBR but displayed a biomedical interpretation for CBR implementation. This demonstrated that many healthcare professionals did not understand that CBR requires various approaches and involves multiple stakeholders.^19,29^ As much as CBR was initially introduced as a health and rehabilitation strategy to increase access to services for PWDs, it has since shifted from a concept purely of health and rehabilitation strategy into a more community-oriented approach.^30^

Rehabilitation is one aspect of CBR, whilst advocacy, community mobilisation, livelihoods, self-help and social dimensions are other essential priorities in CBR. There needs to be a move away from an individual or medical perspective to acknowledge the social and rights-based approach whilst fostering community and social change for inclusion of PWDs.^19,29^ The integration of rehabilitation services into a PHC approach to enhance CBR is regarded as an excellent move to improve the delivery of rehabilitation services whilst empowering the community to take ownership of their health and well-being.^12,31^

Community-based rehabilitation implementation requires a multisectoral, multidisciplinary team approach. In this study, the healthcare professionals had identified the CHWs as the link and liaisons who can play a central role in the success of CBR. Although no other key stakeholder was identified for this pivotal role, there was no mention of training for the CHWs. Whilst CHWs can be used to bridge the gaps in the sustainability of CBR, these cadres of healthcare workers need to be trained and developed to be well-resourced people, who will work together with PWDs and their families to identify and reflect problems, whilst collaborating to find novel solutions within community settings.^29^ Recent evidence refers to such cadres as community-based rehabilitation workers (CRWs) or community-based disability workers (CWDs).^32^ Part of CRW/CWD training includes acquiring skills in facilitation as they often act as the liaison between PWDs, their families, community leaders and professionals.^33,34^ It is further recommended that CRWs/CWDs belong to the communities they serve, as their role involves understanding of community dynamics, identifying gatekeepers to implementing CBR projects, and providing deep insight into cultural and traditional nuances.^35^

The availability of human resources plays a crucial role in ensuring the implementation and sustainability of CBR programmes. There was a resounding awareness of the current challenges of human resource availability for CBR implementation by healthcare professionals in this study. Interestingly, in South Africa, the average number of rehabilitation professionals (occupational therapists, physiotherapists and psychologists) per 100 000 people ranges between 2.6 and 2.8.^36^ The cost related to training and employing these professionals is high. Community-based rehabilitation is an intervention that has moved from professionally centred institutions to the homes and communities of PWDs and where it is carried out by minimally trained people, such as CRWs/CWDs, families and other community members, thereby reducing the financial costs.^34,37^ Therefore, task shifting becomes imperative for effective implementation.^38^ Task shifting was not well understood in our study, as healthcare professionals considered CBR as added pressure requiring resources from a healthcare system that is already strained. This further outlines the lack of CBR training and understanding amongst healthcare professionals. The shortage of human resources is receiving attention and attempts are continually being made to address it. However, the needs of PWDs and their families are often forgotten. The balance between human resource availability and understanding and addressing the needs and concerns of PWDs is an essential factor that is also required for equilibrium in CBR.^10,29,38^

The World Health Organization (WHO) developed CBR guidelines to assist member states in developing their policies informed by their context.^3^ Many countries, including South Africa, have developed policies and legislative acts specific for people with disabilities; however, the translation of these policies into practice has been problematic.^39^ Participants in this study recognised the availability of policies related to CBR; however, they highlighted challenges of translating such policies into everyday practice. The lack of policy translation into practice has been associated with the failure of managers to develop appropriate programmes for CBR, thus contributing further to inadequate allocation of resources, for example, an insufficient budget for assistive devices.^11^

Continual education and training of stakeholders, including healthcare professionals, are crucial for CBR implementation, just as excellent communication and collaboration are critical for the successful roll-out of CBR projects.^40^ In this study, healthcare professionals believed that they were inadequately trained on CBR and its implementation and that it has remained a concept learned in tertiary institutions. Additionally, a few healthcare professionals in this study referred to CBR as a new concept that was not explored at the tertiary level. Other studies have evidenced that barriers for accessing services by PWDs are directly linked to poorly trained healthcare professionals and stakeholders.^31,41^ The training of people involved with CBR and its roll-out is a necessity and should include, amongst others, disability-related technical skills and disability management skills.^11,21^ The training programme needs to be designed and implemented together with PWDs, whilst the universities should incorporate the training into their existing programmes. Gaps in professional education should be identified and addressed, possibly through continual professional development.^42^

Healthcare professionals fear for their safety whilst doing community work. Incidents of crime have been reported in healthcare facilities, where healthcare professionals have been victims. According to a September 2019 Health E-News report, about 35 healthcare facilities in Gauteng Province have been dubbed as ‘crime hotspots’ because of the number of crimes and criminal activities that took place within these healthcare facilities.^43^ Furthermore, various news articles have reported cases of government vehicle theft and hijacking in KZN from multiple departments, including health and agriculture.^44,45,46^ These incidents of crime have compromised the health and safety of healthcare professionals. Therefore, the existence of crime in a community was identified by healthcare workers as a barrier that negatively affects their availability and willingness to provide healthcare services. However, whilst healthcare professionals experience fear for their safety, it should be noted that PWDs are also on the receiving end of crime and poor access to services. This problem seems to be worse in rural areas, where PWDs have to travel many kilometres on foot and at times, often cross rivers to access services.^11^ Yet again, a multisectoral approach to CBR should include departments that are responsible for safety and security of healthcare workers and PWDs to address and mitigate such concerns. Community-based rehabilitation and the needs of PWDs cut across all sectors, such as health, education, social, justice and labour. One sector alone cannot address or respond to the needs of PWDs comprehensively, but each sector needs to collaborate with the others for the betterment and livelihoods of PWDs.^17,19,31^ The collaboration of stakeholders leads to improved awareness about the CBR programme, thereby increasing access to services for PWDs in their communities. The collaboration of stakeholders regarding CBR leads to a shift from the institutional service delivery model of rehabilitation to a community-based approach to service delivery.^11,33,34^

The implementation of CBR was not perceived by healthcare workers in this study as an impossible strategy to implement and made pertinent recommendations for the development and sustainability of such programmes. Participants identified improving community awareness, using local resources and collaboration with key community role players as some key strategies that can be adopted in effort to implement CBR. These recommendations are aligned with the WHO guidelines.^3^ Community awareness aids in the acceptance of PWDs and social integration. Self-esteem of PWDs is improved, whilst stigma, discrimination and negative attitudes are minimised.^6^ The use of local resources, including influential people from communities, will aid in ensuring that CBR programmes are not all one-size fit all but are specific to each community needs and challenges, thereby allowing the community to take ownership of CBR programmes. These recommendations by healthcare professionals are a positive step for the province of KwaZulu-Natal in developing feasible approaches in keeping with WHO guidelines on CBR strategies.^16,40^

It is worth noting that the absence of nurses, in this study, is considered a limitation, as nurses play a crucial role in healthcare delivery. With new reforms in the South African healthcare system and the introduction of family health teams, through PHC re-engineering, the interaction of nurses with people with disabilities and their families has increased. Therefore, their involvement in this study would have been beneficial.

## Conclusion

Continual promotion of, as well as education and training on, CBR for healthcare professionals was understood as an imperative for the development and roll-out of CBR programmes in South African communities. The CBR approach and strategy to rehabilitation have been understood to be complex.

An understanding of the definition and purpose of CBR will assist healthcare professionals to unpack the complex nature of CBR more appropriately, and this is expected to facilitate the development of programmes that will respond to the needs of KwaZulu-Natal. An understanding of CBR should lead to clearly defined roles for the stakeholders responsible for the successful implementation of CBR.

Healthcare professionals also believed that a multisectoral approach to CBR is vital for successful implementation of programmes. Furthermore, good communication amongst stakeholders about CBR programmes is a vital element for ensuring social inclusion, quality of life and access to services for PWDs.

## References

[1] ILO, UNESCO WHO CBR: A strategy for rehabilitation, equalization of opportunities, poverty reduction and social inclusion of people with disabilities. Int J Epidemiol. [serial online] 2004 [cited 2019 Mar 26];29(5):956 Available from: http://www.ncbi.nlm.nih.gov/pubmed/11034985

[2] World Health Organization (WHO) aWorld report on disability 2011 [homepage on the Internet]. World Health Organization, World Bank; 2011 [cited 2019 Jul 11]. Available from: http://www.ncbi.nlm.nih.gov/pubmed/22726850

[3] World Health Organization (WHO) CBR guidelines. Geneva: World Health Organization; 2010.

[4] Wickenden M, Mulligan D, Fefoame GO, Katende P Stakeholder consultations on community-based rehabilitation guidelines in Ghana and Uganda. Afr J Disabil. 2012;1(1):1–10. 10.4102/ajod.v1i1.1 28729971 PMC5442565

[5] Iemmi V, Gibson L, Blanchet K, et al Community-based rehabilitation for people with disabilities in low- and middle-income countries: A systematic Review [homepage on the Internet]. 2015 [cited 2018 Nov 08]. Available from: https://campbellcollaboration.org/library/community-based-rehabilitation-people-with-disabilities.html

[6] World Health Organization (WHO) Better health for all people with disability. Geneva: World Health Organization; 2015.

[7] Sida Disability rights in sub-Saharan Africa [homepage on the Internet]. 2015 [cited 2019 Nov 13]. Available from: http://www.sida.se/globalassets/sida/eng/partners/human-rights-based-approach/disability/rights-of-persons-with-disabilities-sub-saharan-africa.pdf

[8] Statistics South Africa Profile of persons with disabilities in South Africa, census 2011. Tshwane: Stats SA- South African Government; 2014.

[9] Statistics South Africa Community survey 2016-profiling the Socio-Ecomomic Status and Arrangement of People with Disabilities in South Africa [Internet]. Tshwane: Stats SA - South Africa Government; 2016 Available from: http://cs2016.statssa.gov.za/wp-content/uploads/2018/07/CS-2016-Disability-Report_-03-01-232016.pdf

[10] Gilmore B, MacLachlan M, McVeigh J, et al A study of human resource competencies required to implement community rehabilitation in less resourced settings. Hum Resour Health. 2017;15(1):70 10.1186/s12960-017-0240-1 28938909 PMC5610467

[11] Cayetano RDA, Elkins J Community-based rehabilitation services in low and middle-income countries in the Asia-Pacific region: Successes and challenges in the implementation of the CBR matrix. Disabil CBR Incl Dev. 2016;27(2):112–127.

[12] Govender P, Naidoo D, Bricknell K, Ayob Z, Message H, Njoko S ‘No one prepared me to go home’: Cerebrovascular accident survivors’ experiences of community reintegration in a peri-urban context. Afr J Prim Health Care Fam Med. 2019;11(1):1–8. 10.4102/phcfm.v11i1.1806 PMC6489154 31038341

[13] Crishna B What is community-based rehabilitation? A view from experience. Child Care Health Dev. 1999;25(1):27–35. 10.1046/j.1365-2214.1999.00087.x 9921419

[14] Fisher P, Goodley D The linear medical model of disability: Mothers of disabled babies resist with counter-narratives. Sociol Heal Illn. 2007;29(1):66–81. 10.1111/j.1467-9566.2007.00518.x 17286706

[15] Rule S, Lorenzo T, Wolmerans M Community based rehabilitation: New challenges. In Disability and social change: A South African agenda [homepage on the Internet]. 2006; p. 273–290 [cited 2019 Nov 12]. Available from: http://www.hsrcpress.ac.za/product.php?productid=2151&freedownload=1

[16] Lang R Community-based rehabilitation and health professional practice: Developmental opportunities and challenges in the global North and South. Disabil Rehabil. 2011;33(2):165–173. 10.3109/09638288.2010.487923 20500051

[17] Morita H, Yasuhara K, Ogawa R, Hatanaka H Factors impeding the advancement of community based rehabilitation (CBR): Degree of understanding of professionals about CBR. J Phys Ther Sci. 2013;25(4):413–423. 10.1589/jpts.25.413

[18] Chappell P, Johannsmeier C The impact of community based rehabilitation as implemented by community rehabilitation facilitators on people with disabilities, their families and communities within South Africa. Disabil Rehabil. 2009;31(1):7–13. 10.1080/09638280802280429 19194807

[19] Rule S, Roberts A, McLaren P, Philpott S South African stakeholders’ knowledge of community-based rehabilitation. Afr J Disabil. 2019;8(1):1–12. 10.4102/ajod.v8i0.484 PMC6779963 31616621

[20] South African Department of Health National rehabilitation policy. Tshwane: South African Government; 2000, p. 1025–1045.

[21] South African Department of Health Framework and strategy for rehabilitation services 2015–2020. Tshwane: S.A. Government; 2015, p. 1–26.

[22] Rule S, Lorenzo T, Wolmarans M Community-based rehabilitation: New challenges. Cape Town: HSRC Press; 2004, p. 273–290.

[23] Creswell JW Research design: Qualitative, quantitative and mixed methods approach. 3rd ed. Volume 8 Muqarnas. Thousand Oaks: SAGE Publications Inc; 2009; p. 94–102.

[24] Govender P, Chetty V, Naidoo D, Pefile N Integrated decentralized training for health professions education at the university of KwaZulu-Natal, South Africa: Protocol for the i-dect project. J Med Internet Res. 2018;20(1). 10.2196/resprot.7551 PMC5806004 29371175

[25] Saldaña J The coding manual for qualitative researchers [homepage on the Internet]. 2nd ed. Sage; 2013; p. 1–329. [cited 2019 Jan 29]. Available from: https://www.cambridge.org/core/product/identifier/CBO9781107415324A009/type/book_part.

[26] WHO International consultation to review community-based rehabilitation (CBR) [homepage on the Internet]. 2003; p. 1–20. [cited 2018 Nov 18]. Available from: http://apps.who.int/iris/bitstream/10665/68466/1/WHO_DAR_03.2.pdf

[27] World Health Organization (WHO) Community home-based care training [homepage on the Internet]. Searo; 2017; [cited 2018 Nov 8]. Available from: http://www.searo.who.int/myanmar/areas/hivaidschbctraining/en/

[28] Hanass-Hancock J, Nixon SA The fields of HIV and disability: Past, present and future. J Int AIDS Soc. 2009;2(1):3. 10.1186/1758-2652-12-28 PMC2788341 19900282

[29] Kuipers P, Cornielje H Alternative responses to the human resource challenge for CBR. Asia Pacific Disabil Rehabil J. 2012;23(4):17–23. 10.5463/DCID.v23i4.176

[30] WHO, SHIA Community-based rehabilitation as we have experienced it …voices of persons with disabilities in Ghana, Guyana and Nepal [homepage on the Internet]. 2002; p 44 [cited 2018 Nov 08]. Available from: https://whqlibdoc.who.int/publications/9241590440.pdf

[31] Karthikeyan P, Ramalingam KP Physiotherapy training to enhance community-based rehabilitation services in Papua New Guinea: An educational perspective. Asia Pacific Disabil Rehabil J. 2014;25(1):82–94. 10.5463/DCID.v25i1.259

[32] Van Pletzen E, Booyens M, Lorenzo T An exploratory analysis of community-based disability workers’ potential to alleviate poverty and promote social inclusion of people with disabilities in three Southern African countries. Disabil Soc. 2014;29(10):1524–1539. 10.1080/09687599.2014.958131

[33] Biggeri M, Deepak S, Mauro V, Trani JF, Kumar J, Ramasamy P Do community-based rehabilitation programmes promote the participation of persons with disabilities? A case control study from Mandya District, in India. Disabil Rehabil. 2014;36(18):1508–1517. 10.3109/09638288.2013.823244 23944177

[34] Deepak S, Biggeri M, Mauro V, Kumar J, Griffo G Impact of community-based rehabilitation on persons with different disabilities. Asia Pacific Disabil Rehabil J. 2013;24(4):5–23.

[35] Neille J, Penn C Beyond physical access: A qualitative analysis into the barriers to policy implementation and service provision experienced by persons with disabilities living in a rural context. Rural Remote Health. 2015;15(3):3332. 26268958

[36] Department of Social Development Elements of the financial and economic costs of disability to households in South Africa: A pilot study. Department of Social Development; 2015.

[37] Bongo PP, Dziruni G, Muzenda-Mudavanhu C The effectiveness of community-based rehabilitation as a strategy for improving quality of life and disaster resilience for children with disability in rural Zimbabwe. Jamba. 2018;10(1):442 10.4102/jamba.v10i1.442 29955257 PMC6014043

[38] Chetty V, Hanass-Hancock J A rehabilitation model as key to comprehensive care in the era of HIV as a chronic disease in South Africa. AIDS Care. 2016;28(Suppl 1):132–139. 10.1080/09540121.2016.1146204 27002771 PMC4828600

[39] M’kumbuzi VRP, Myezwa H Conceptualisation of community-based rehabilitation in Southern Africa: A systematic review. S Afr J Physiother. 2016;72(1):1–8. 10.4102/sajp.v72i1.301 PMC6093098 30135885

[40] Lightfoot E Community-based rehabilitation A rapidly growing method for supporting people with disabilities. Int Soc Work. 2004;47(4):455–468. 10.1177/0020872804046253

[41] Mijnarends D, Pham D, Swaans K, Van Brakel WH, Wright P Sustainability criteria for CBR programmes – Two case studies of provincial programmes in Vietnam. Disabil CBR Incl Dev. 2011;22(2):3–21. 10.5463/dcid.v22i2.54

[42] Sachdeva AK Continuing professional development in the twenty-first century. J Contin Educ Health Prof. 2016;36(Suppl 1):S8–S13. 10.1097/CEH.0000000000000107 27584081

[43] Msomi N About 35 hospitals and clinics are crime hotspots in Gauteng. Newslth E-News 2019 [cited no date]. Available from: https://health-e.org.za/2019/09/20/about-35-hospitals-and-clinics-are-crime-hotspots-in-gauteng/

[44] Ndaliso C Hijack-targeted state vehicles may be insured. 2016 [cited no date]. Available from: https://www.iol.co.za/news/hijack-targeted-state-vehicles-may-be-insured-2065999

[45] Two nabbed over hijacked KZN government vehicles The Mercury 2017 [cited no date]. Available from: https://www.iol.co.za/news/two-nabbed-over-hijacked-kzn-governmentvehicles-7451431

[46] R50k reward offered following theft of eight KZN state vehicles The Citizen 2020 [cited no date]. Available from: https://citizen.co.za/news/south-africa/crime/2240762/r50k-rewardoffered-following-theft-of-eight-kzn-state-vehicles/

